# Mild-Intensity UV-A Radiation Applied Over a Long Duration Can Improve the Growth and Phenolic Contents of Sweet Basil

**DOI:** 10.3389/fpls.2022.858433

**Published:** 2022-04-18

**Authors:** Seonghwan Kang, Jo Eun Kim, Shuyang Zhen, Jongyun Kim

**Affiliations:** ^1^Department of Horticultural Sciences, Texas A&M University, College Station, TX, United States; ^2^Department of Horticultural Biotechnology, Korea University, Seoul, South Korea; ^3^Department of Plant Biotechnology, Korea University, Seoul, South Korea

**Keywords:** *Ocimum basilicum*, UV-A, controlled environment agriculture, antioxidant capacity, biomass, phenolic contents

## Abstract

UV-A radiation (320–400 nm) is an abiotic stressor that may be used to enhance the production of beneficial secondary metabolites in crops such as leafy vegetables. However, tradeoffs between enhanced phytochemical contents and overall growth/yield reductions have been reported. The responses varied depending on the UV-A intensity, spectral peak, exposure time, species, and varieties. We quantified the changes in growth, morphology, photosynthesis, and phenolic contents of sweet basil grown under a base red/blue/green LED light with four supplemental UV-A intensity treatments (0, 10, 20, and 30 W·m^−2^) in an indoor environment over 14 days. The objective was to determine whether UV-A radiation could be utilized to improve both yield and quality of high-value sweet basil in a controlled production environment. Biomass harvested at 14 days after treatment (DAT) was highest under mild-intensity UV-A treatment of 10 W·m^−2^ and lowest under high-intensity UV-A treatment of 30 W·m^−2^. The total leaf area and the number of leaves were significantly lower under the 30 W·m^−2^ treatment than under the 10 and 20 W·m^−2^ treatments at 14 DAT. The maximum quantum efficiency of photosystem II (PSII) for photochemistry (*F_v_/F_m_*) showed a gradual decrease under the 20 and 30 W·m^−2^ treatments from 3 to 14 DAT, whereas *F_v_/F_m_* remained relatively constant under the 0 and 10 W·m^−2^ treatments over the entire 14 days. The leaf net photosynthesis rate showed a significant decrease of 17.4% in the 30 W·m^−2^ treatment compared to that in the 10 W·m^−2^ treatment at 14 DAT. Phenolic contents (PAL enzyme activity, total phenolic concentration, and antioxidant capacity) were the highest under the 20 W·m^−2^ treatment, followed by the 10, 30, and 0 W·m^−2^ treatments. Overall, our results indicate that the biomass production and accumulation of beneficial phenolic compounds in sweet basil varied depending on the intensity and duration of UV-A application. Mild UV-A radiation (10–20 W·m^−2^) can be a beneficial stressor to improve sweet basil yield and quality over relatively long-term cultivation.

## Introduction

In horticultural crop production, abiotic stresses, such as drought, cold temperature, and UV radiation, have been used to improve crop morphological traits and enhance the production of beneficial phytochemicals. Plants often accumulate secondary metabolites as a defense mechanism against damage caused by increased reactive oxygen species (ROS) formation under abiotic stresses such as drought and temperature extremes (Zhang and Kirkham, 1994; Dixon and Paiva, 1995; Shah et al., 2001; Gill and Tuteja, 2010; Sharma et al., 2012; Verma and Shukla, 2015). The level of ROS formation depends on the intensity and duration of abiotic stresses, cultivation conditions, and plant variety (Miller et al., 2010; Nikolaeva et al., 2010). Plants grown under high abiotic stresses can suffer from damage by excessive ROS accumulation, such as lipid peroxidation of cell membranes, DNA and RNA damage, and protein oxidation (Imlay, 2003). Under mild abiotic stresses, plants may be able to effectively reduce ROS and oxidative stress levels by synthesizing enzymatic (e.g., superoxide dismutase and ascorbate peroxidase) and non-enzymatic (e.g., ascorbate, glutathione, carotenoids, and phenolics) antioxidants that act as ROS scavengers (Noctor and Foyer, 1998). The contents of total phenols and flavonoids, which are common antioxidants, may increase to prevent cell damage by ROS (Garg and Manchanda, 2009). Lee et al. (2019a) reported that non-enzymatic antioxidants produced by plants have anti-aging benefits and could improve human health by reducing the risk of diseases, including cancer and chronic cardiovascular diseases. The potential benefits of varying levels of abiotic stress on plant growth and production of phytochemicals with antioxidant properties have been actively studied (Akula and Ravishankar, 2011; Boo et al., 2011; Lee et al., 2019a; Nam et al., 2020).

With growing interest in environmentally controlled indoor plant production, there has been a rapidly increasing number of studies investigating the effects of narrow-spectrum light-emitting diode (LED) lighting on plant growth and morphological and physiological responses (Brazaityte et al., 2015; Chen et al., 2019; Lee et al., 2019b; Zhen and Bugbee, 2020; Zhen et al., 2022). Ultraviolet (UV) has been used as an abiotic stressor to stimulate plant phytochemical production in high-value plants. Apart from phytochemical synthesis, UV radiation can affect a wide range of plant responses, and its effects depend on the wavelength, light intensity, and exposure period (Loconsole and Santamaria, 2021).

UV radiation is classified into three sub-categories: UV-C (100–280 nm), UV-B (280–320 nm), and UV-A (320–400 nm). The ozone layer completely absorbs UV-C radiation from sunlight. In contrast, UV-A and UV-B reach the Earth’s surface. UV-B photons, which contain higher energy than UV-A photons, are known to induce DNA damage, impair various cellular processes, and reduce leaf expansion and crop growth when applied at high intensity or over long durations (Dou et al., 2019).

The effects of lower-energy UV-A radiation have not been studied in depth. Both beneficial and detrimental effects on plant growth and phytochemical production have been observed, depending on the dosage of UV-A applied. Lee et al. (2019b) reported that UV-A radiation (peak at 385 nm) applied at 30 W·m^−2^ for a relatively short period (5 days) increased both the growth and the content of phenolic compounds in kale. Chen et al. (2019) found that lettuce grown under UV-A for 13 days (applied at 10, 20, or 30 μmol·m^−2^·s^−1^; peak at 365 nm) had higher fresh and dry weight, leaf area, and antioxidant content than those grown without UV-A application. Zhang et al. (2020) similarly showed that tomato plants grown under a mixture of red light (215 μmol·m^−2^·s^−1^) and UV-A (35 μmol·m^−2^·s^−1^) showed improved growth and higher total flavonoid content than those grown under red light only. In contrast, other studies found that UV-A radiation (peak at 365 nm) applied at 13.8–15 W·m^−2^ in spinach and pea can increase ROS production, and subsequently, a reduction in photosynthetic electron transport rate and inhibition of photosynthesis (Vass et al., 2002; Ivanova et al., 2008; Loconsole and Santamaria, 2021).

The contrasting responses to UV-A radiation are most likely due to differences in the intensity, duration, or wavelength of the UV-A applied as well as species sensitivity. The potential tradeoffs between reduced crop yield and improved quality attributes in response to various UV intensities must be carefully considered. Species-specific responses need to be characterized to enhance growth and phytochemical production with UV-A treatments.

The objective of this study was to quantify the morphological and physiological responses and changes in the phenolic content of sweet basil under a range of UV-A light intensities (from 0 to 30 W·m^−2^) over relatively long-term crop cultivation in an indoor production system using LEDs. This information can be used to develop an improved lighting protocol for producing high-quality sweet basil, a high-value crop, in controlled environments.

## Materials and Methods

### Plant Material and Growing Conditions

Sweet basil seeds (*Ocimum basilicum* L. “Sweet Basil”; Asia Seed Co., Seoul, Korea) were sown in two 128-cell plug trays with a germinating substrate containing 80% peat and 20% perlite (Sunshine Mix#5, Sun Gro Horticulture, Agawam, MA, United States). After a month, 175 seedlings with similar growth were selected and transplanted to round plastic pots (440 ml; 10 cm in diameter) filled with a soilless substrate containing 70% peat and 30% perlite (Sunshine Mix #4, Sun Gro Horticulture) mixed with a controlled release fertilizer (14 N-6.1P-11.6 K; 14-14-14 Multicote 6, Haifa Chemicals, Israel) at a rate of 4 g·L^−1^. After transplanting, all plants were transferred to an environmentally controlled walk-in growth room with a combination of red (R), blue (B), and green (G) light provided by light-emitting diodes (LEDs; described in more detail below). Plants were allowed to acclimate for 1 week prior to the initiation of UV-A light treatments. The environmental conditions inside the growth room were recorded using a data logger (CR1000; Campbell Scientific, Logan, UT, United States). The temperature was maintained at 25.0 ± 0.5°C (mean ± SD), relative humidity was 68.4 ± 2.6%, and CO_2_ concentration was enriched to 910.2 ± 15.0 μmol·mol^−1^.

Prior to the treatment, 112 uniform plants at the third leaf stage were selected (4 treatments × 7 plants (sub-samples) per treatment × 4 replicates; 16 total experimental units). The base height of the containers was adjusted regularly to minimize changes in light intensity at the canopy level. Plants were irrigated using a soil moisture sensor-automated irrigation system similar to the one described by Nemali and van Iersel (2006). The irrigation system maintained the substrate volumetric water content of each container at 0.5 m^3^·m^−3^. An additional eight uniform basil plants were used to measure the initial physiological parameters, phenolic compound content, and fresh/dry mass.

### Light Treatments

**Figure 1:**
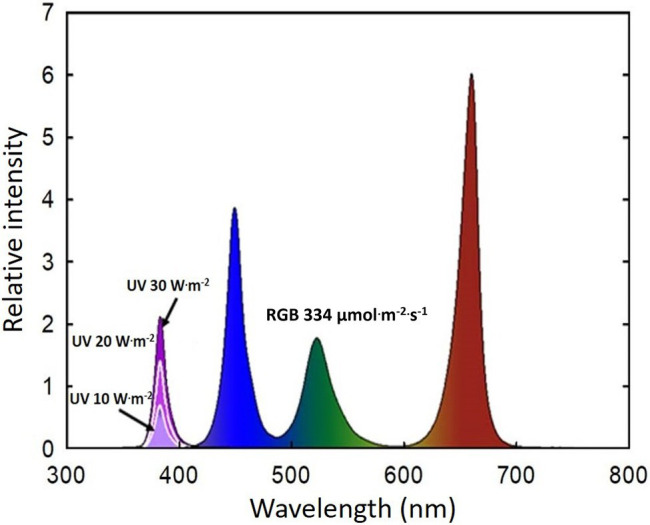
Spectral distribution of four UV-A light intensity treatments (0 W·m^−2^, 10 W·m^−2^, 20 W·m^−2^, and 30 W·m^−2^, peak at 385 nm) with RGB LEDs (red, green, and blue peaks at 664, 524, and 451 nm, respectively).

During the one-week acclimation period, basil plants were placed under a combination of RGB LEDs (ES LEDs, Seoul, Korea) without UV-A treatments. The B (400–500 nm):G (501–600 nm):R (601–700 nm) ratio was 30.9:21.6:47.5 (Figure 1), mimicking the spectral composition of sunlight. The light intensity at the top of the plant canopy was 333.9 ± 1.0 μmol·m^−2^·s^−1^. UV-A light (365–399 nm, peak at 385 nm) at 0, 10, 20, and 30 W·m^−2^ (photon flux density of 0, 35, 65, and 97 μmol·m^−2^·s^−1^, respectively) was added to the background RGB light for 14 days. The UV light was provided by UV LEDs built into the same LED system (ES LEDs). The photoperiod was 16 h day/8 h night. The spectral distribution was measured at the top of the plant canopy (15 cm from the lighting source) using a spectroradiometer (PS300; Apogee Instruments, Logan, UT, United States). Because the light distribution of the LEDs was not perfectly uniform, plants within each experimental unit were systemically rotated every day to minimize any effects caused by the small differences in spectral ratio.

### Growth and Photosynthetic Parameters

At the onset of the experiment, the initial growth and photosynthetic parameters were determined in eight plants [0 days after treatment (DAT)]. Two plants per experimental unit were harvested at 3, 7, and 14 DAT to quantify the changes in growth, photosynthetic parameters, and secondary metabolite contents of basils in response to the UV-A treatments. Plant height, width, and fresh shoot were measured at each harvest. The number of leaves was counted, and the individual and total leaf area were measured using a leaf area meter (LI-3000, LI-COR, Lincoln, NE, United States). Leaf size was calculated as the average of the three largest leaves of each plant. Leaf chlorophyll content was estimated at the midpoint of each uppermost fully expanded leaf blade with an average of three measurements per plant using a soil plant analysis development (SPAD) chlorophyll meter (SPAD-502, Minolta Corporation Ltd., Osaka, Japan). Shoot dry weight was determined after the shoots were dried for 5 days at 80°C in a drying oven.

The photosynthetic rate, transpiration rate, and stomatal conductance were measured on the uppermost fully expanded leaves using a portable photosynthesis system (CIRAS-3, PP System, Amesbury, MA, United States) equipped with a CIRAS LED irradiation module around noon. Cuvette environments were set the same as the environments of the growth room (temperature at 25°C, RH of 67%, CO_2_ concentration of 900 ppm, and PPFD of 334 μmol·m^−2^·s^−1^). The maximum quantum efficiency of photosystem II photochemistry (*F_v_/F_m_*) was measured on the same leaves using a chlorophyll fluorescence meter (Junior PAM, Heinz Walz GmbH, Effeltrich, Germany) to detect any stress or photoinhibition induced by the UV-A light treatments. After dark-adapting the leaves for 20 min, the maximum variable fluorescence (*F_v_*) and minimum fluorescence (*F_o_*) were obtained by applying a saturating light pulse. The maximum PSII quantum yield (*F_v_/F_m_*) was calculated as *F_v_/F_m_* = (*F_m_−F_o_*)/*F_m_* (Maxwell and Johnson, 2000).

### Total Phenolic Concentration

The total phenolic concentration was determined according to (Ainsworth and Gillespie, 2007). Sweet basil has an opposite leaf arrangement, with two leaves per node. One of the two uppermost fully expanded leaves was collected for the phenolic content measurements during each harvest. Approximately 0.2 g of each fresh basil leaf was immediately frozen in liquid nitrogen and stored at −70°C before total phenolic concentration analysis. Samples were ground in liquid nitrogen with 3 ml of 80% acetone using a mortar and pestle and then incubated at 4°C for 16 h in the dark. Following centrifugation at 3,000 × *g* for 20 min, 100 μl of the supernatant was used for analysis. The supernatant was mixed with 270 μl of H_2_O, 1.5 ml of 10% Folin–Ciocalteu reagent (Sigma-Aldrich, St Louis, MO, United States), and 1.2 ml of 7.5% Na_2_CO_3_. After vortexing for 20 s, the mixtures were incubated at 45°C in a water bath for 15 min. The absorbance was measured at 765 nm using a spectrophotometer (Super Aquarius 9,000 Series, Suwon, Korea). The concentrations of the standard (gallic acid) used for the calibration curve were the following: 0, 0.0625, 0.125, 0.25, 0.5, and 1 mg/ml. The total phenolic concentration was expressed as the milligrams of gallic acid equivalents per gram fresh weight (mg GAE·g^−1^ FW).

### Antioxidant Capacity

Antioxidant capacity was quantified as the ability of antioxidants to scavenge the 2,2-azino-bis (3-ethylbenzothiazoline-6-sulfonic acid) radical cation (ABTS; Sigma-Aldrich, St. Louis, MO, United States), following the method described by (Miller and Rice-Evans, 1996). Samples (0.2 g) were collected from the leaves to measure the total phenolic compounds. Each sample was immediately frozen in liquid nitrogen and stored at −70°C prior to analysis. The samples were extracted with 3 ml of 80% acetone, incubated at −20°C for 16 h in the dark, and then centrifuged at 3,000*g* for 2 min. Subsequently, 0.1 ml of the supernatant was diluted with 0.9 ml of 80% acetone. Approximately 0.4 g of MnO_2_ was mixed with 20 ml of ABTS (Sigma-Aldrich) stock solution (2.5 mmol·L^−1^) and stirred for 30 min to generate the ABTS radical cation (ABTS*). The ABTS* solution was first filtered using a filter paper (type 5 B; Advantec, Tokyo, Japan) to eliminate excess MnO_2_ and then using a 0.2 μm syringe filter (PVDF syringe filter; Whatman). To obtain an ABTS* solution with an absorbance of 0.7 ± 0.02 at 730 nm, 5 mm phosphate-buffered saline [PBS; pH 7.4, ionic strength (150 mmol L^−1^ NaCl)] was used for dilution. The sample extract (150 μl) was added to 1.5 ml of ABTS* solution and vortexed for 20 s. The absorbance of the mixture was measured using a spectrophotometer exactly 1 min after initial mixing. The concentration of the standard (trolox) used for the calibration curve ranged from 0 to 200 μm. The antioxidant capacity was expressed using millimoles Trolox (6-hydroxy-2, 5, 7, 8-tetramethylchroman-2-carboxylic acid; Sigma-Aldrich) equivalent per gram of fresh weight (mmol TEAC·g^−1^ FW) of basil leaves.

### Phenylalanine-Ammonia Lyase Enzyme Activity

Phenylalanine-ammonia lyase (PAL) activity was analyzed according to Boo et al. (2011). Samples (0.5 g) were collected from the same leaves used to analyze total phenolic concentration and antioxidant capacity and were stored at −80°C prior to PAL activity analysis. The sample was ground in liquid nitrogen and extracted with 10 ml of 25 mm borate buffer (pH 8.8) and 2 ml of 3 mm β-mercaptoethanol (Sigma-Aldrich, St. Louis, MO, United States). The extract was centrifuged at 2,000*g* for 20 min. After centrifugation, the supernatant was mixed with 2.5 mm borate buffer (pH 8.8) and 2.5 ml of 10 mm L-phenylalanine (Sigma-Aldrich, St. Louis, MO, United States) and incubated at 40°C for 2 h. The addition of 0.1 ml HCl stopped the reaction, and the absorbance was measured at 290 nm using a spectrophotometer. A standard curve using trans-cinnamic acid (calibrated at 0, 0.0625, 0.125, 0.25, 0.5, and 1 mm; Sigma-Aldrich, St. Louis, MO, United States) equivalent in mmol per hour per gram of fresh weight (mmol trans-cinnamic acid· h^−1^·g^−1^ FW) was used to determine PAL activity.

### Experimental Design and Statistical Analysis of Data

The experiment was conducted as a randomized complete block design with four blocks and four treatments. The data analysis was conducted using three-way ANOVA in statistical analysis system (SAS 9.4, SAS Institute, Cary, NC, United States) to test the effects of treatment (TRT), block, and time effect (days after treatment; DAT). Tukey’s honestly significant difference test was used to compare the means among the treatments (PROC GLM; SAS 9.4, SAS Institute), with statistical significance set at *p* < 0.05.

## Results

### Changes in Growth Parameters in Response to UV-A Light Intensities

**Figure 2:**
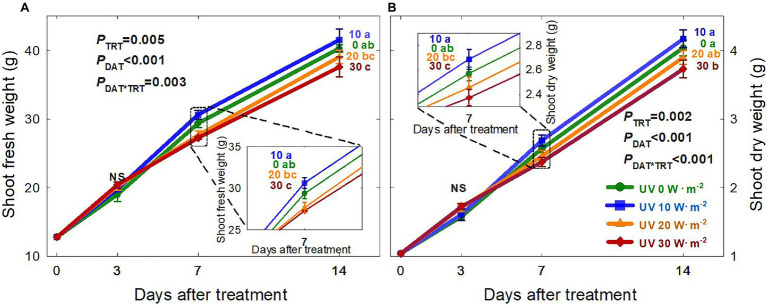
Shoot fresh weight **(A)** and shoot dry weight **(B)** of *Ocimum basilicum* under different UV-A light intensities on various days after treatment (0, 10, 20, and 30 W·m^−2^; peak at 385 nm) with RGB LEDs (red, green, and blue peaks at 664, 524, and 451 nm, respectively). Data points represent mean ± SE (*n* = 8; 2 plants per treatment × 4 replications). Different letters indicate a significant UV-A treatment difference at *p* < 0.05 after Tukey’s HSD test. *p*-values of the three-way ANOVA analysis for the UV-A treatments (TRT), time effect (days after treatment; DAT), and the interactive effects between UV-A treatments and time (DAT*TRT) are shown in the figures.

**Figure 3:**
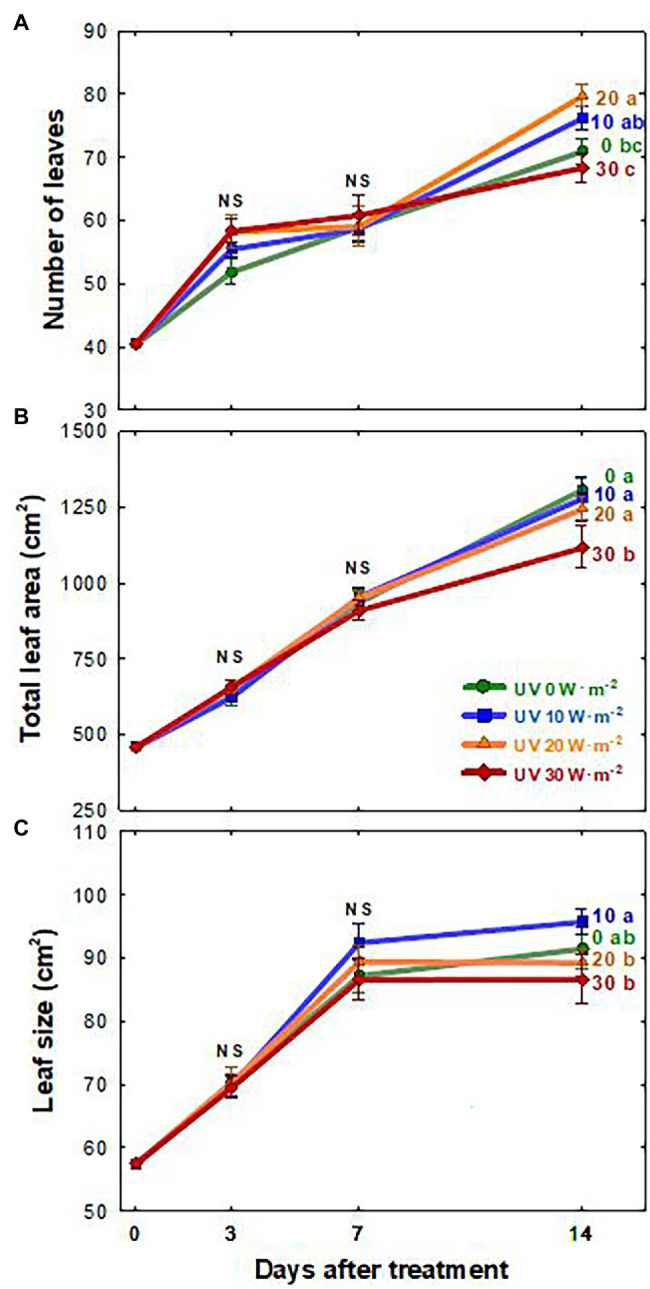
Number of leaves **(A)**, total leaf area **(B)**, and leaf size **(C)** of *Ocimum basilicum* according to different UV-A light intensities (0, 10, 20, and 30 W·m^−2^; peak at 385 nm) with RGB LEDs (red, green, and blue peaks at 664, 524, and 451 nm, respectively). Data points represent mean ± SE (*n* = 8; 2 plants per treatment × 4 replications). Different letters indicate a significant difference at *p* < 0.05 after Tukey’s HSD test.

The growth of basil plants in all four UV-A treatments (0, 10, 20, and 30 W·m^−2^) increased from 0 to 14 DAT (the last day of the experiment). However, there were no significant differences in plant height, width, leaf thickness, and chlorophyll content (as indicated by the SPAD index) among treatments during the experimental period (Supplementary Table S1). Shoot fresh and dry weights were the highest under the mild UV-A treatment (10 W·m^−2^) at both 7 and 14 DAT, whereas plants grown under UV-A intensities of 20 and 30 W·m^−2^ had a reduced biomass production (Figure 2). At the end of the experiment, shoot fresh weight and dry weight of plants under the highest intensity UV-A treatment (30 W·m^−2^) were significantly lower than those in the 0 and 10 W·m^−2^ treatments, with a 10.6% reduction in dry weight compared to that under the 10 W·m^−2^ UV-A treatment. Under the 30 W·m^−2^ treatment, leaf number, total leaf area, and leaf size were significantly reduced by 10.3, 12.6, and 9.5% at 14 DAT, respectively, compared to those in the mild UV-A treatment of 10 W·m^−2^ (Figure 3). Overall, biomass production and total leaf area were reduced under high dosages of UV-A compared to the 0 (control) and 10 (mild) W·m^−2^ UV-A treatments. In contrast, there were no significant differences between plants grown under 0 and 10 W·m^−2^ UV-A treatments.

### Gas Exchange and Chlorophyll Fluorescence

#### Photosynthetic Rate

**Figure 4:**
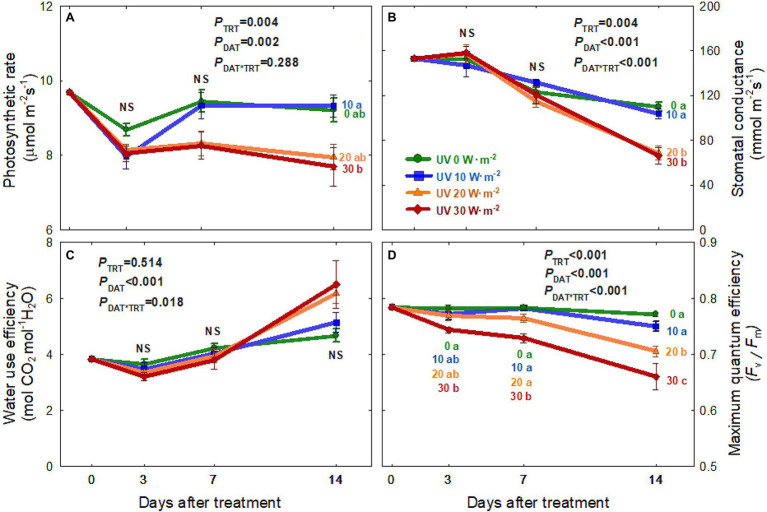
Net photosynthetic rate **(A)**, stomatal conductance **(B)**, water-use efficiency **(C)**, and maximum quantum efficiency of PSII (*F_v_/F_m_*) **(D)** of *Ocimum basilicum* according to different UV-A light intensities (0, 10, 20, and 30 W·m^−2^; peak at 385 nm) with RGB LEDs (red, green, and blue peaks at 664, 524, and 451 nm, respectively) as days after treatment. Data points represent mean ± SE (*n* = 8; 2 plants per treatment × 4 replications). Different letters indicate a significant UV-A treatment difference at *p* < 0.05 after Tukey’s HSD test. *p*-values of the three-way ANOVA analysis for the UV-A treatments (TRT), time effect (days after treatment; DAT), and the interactive effects between UV-A treatments and time (DAT*TRT) are shown in the figures.

The net photosynthetic rate (P_net_) of all four treatments decreased from 0 to 3 DAT (Figure 4A). Plants under the 0 and 10 W·m^−2^ UV-A treatments showed a recovery of P_net_ to pre-treatment levels at 7 DAT. However, there were no significant differences among the four treatments. At 14 DAT, P_net_ showed a significant reduction of 17.4% in the 30 W·m^−2^ UV-A treatment compared to the 10 W·m^−2^ treatment.

#### Stomatal Conductance and Water-Use Efficiency

The stomatal conductance (g_s_) of plants from all four treatments decreased over time, with greater reductions in the two higher intensity UV-A treatments (20 and 30 W·m^−2^; Figure 4B). At 14 DAT, g_s_ under the 20 and 30 W·m^−2^ treatments were 37–40% lower than those under the 0 and 10 W·m^−2^ treatments. However, there were no significant differences in water-use efficiency among the treatments over the experimental period (Figure 4C).

#### Dark-Adapted *F_v_/F_m_*

The maximum quantum efficiency of PSII photochemistry (*F*_v_/*F*_m_) decreased over time in the two higher intensity UV-A treatments (20 and 30 W·m^−2^), with a greater decrease in the 30 W·m^−2^ treatment (Figure 4D). In contrast, *F*_v_/*F*_m_ values were relatively constant in the 0 W·m^−2^ treatment (0.78 ± 0.01; average ± SD) and 10 W·m^−2^ treatment (0.77 ± 0.02) over the experimental period. At 14 DAT, *F*_v_/*F*_m_ was 8.6% lower in the 20 W·m^−2^ UV-A treatment and 17% lower in the 30 W·m^−2^ treatment than in the 0 W·m^−2^ treatment.

### Total Phenolic Concentration, Antioxidant Capacity, and PAL Enzyme Activity

**Figure 5:**
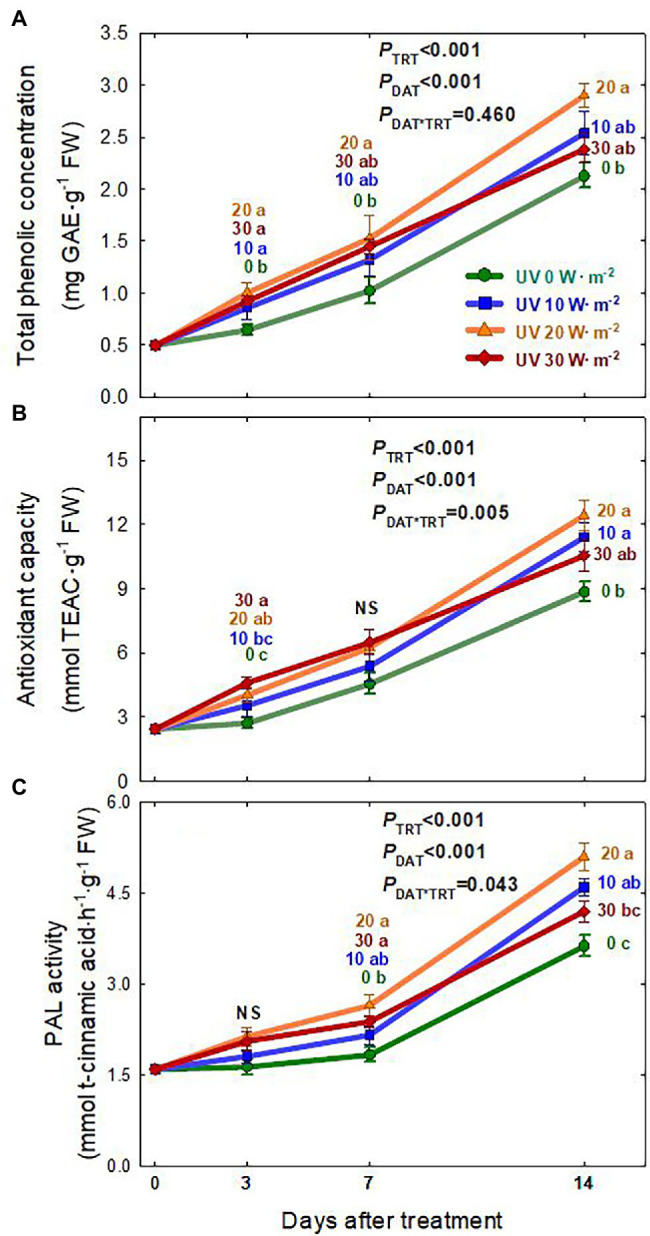
Total phenolic concentration **(A)**, Antioxidant capacity **(B)**, and PAL enzyme activity **(C)** of *Ocimum basilicum* according to different UV-A light intensities at days after treatment (0, 10, 20, and 30 W·m^−2^; peak at 385 nm) with RGB LEDs (red, green, and blue peaks at 664, 524, and 451 nm, respectively). Data points represent mean ± SE (*n* = 8; 2 plants per treatment × 4 replications). Different letters indicate a significant UV-A treatment difference at *p* < 0.05 after Tukey’s HSD test. *p*-values of the three-way ANOVA analysis for the UV-A treatments (TRT), time effect (days after treatment; DAT), and the interactive effects between UV-A treatments and time (DAT*TRT) are shown in the figures.

The total phenolic concentration, antioxidant capacity, and PAL enzyme activity of basil leaves generally increased over the treatment period and showed significant differences among the UV-A treatments (Figure 5). Overall, plants grown under the 20 W·m^−2^ treatment tended to have the highest total phenolic concentration, antioxidant capacity, and PAL enzyme activity, followed by the 10, 30, and 0 W·m^−2^ treatments. At 14 DAT, PAL enzyme activity was 38.4% higher in the 20 W·m^−2^ treatment and 25.4% higher in the 10 W·m^−2^ treatment than in the 0 W·m^−2^ UV-A treatment. The total phenolic concentration was 36.3% higher in the 20 W·m^−2^ treatment than in the 0 W·m^−2^ UV-A treatment, and antioxidant capacities in the 20 and 10 W·m^−2^ treatments increased by 40.5 and 28.8%, respectively, compared to that in the 0 W·m^−2^ UV-A treatment.

## Discussion

### Biomass Production in Response to UV-A Light Intensities

This study showed that mild UV-A application (10 W·m^−2^) improved biomass production. However, higher UV-A intensities (30 W·m^−2^) reduced biomass production of sweet basil after 14 days of treatment (Figure 2). This was consistent with previous findings that the effects of UV-A on biomass production varied depending on the intensity of UV-A and other factors such as the intensity of photosynthetically active radiation photons and plant species/cultivar. Lee et al. (2019b) reported that kale shoot fresh and dry weights were significantly increased after treatment with 30 W·m^−2^ UV-A (peak at 385 nm) for 5 days compared to 0 W·m^−2^ UV-A treatment. A field study indicated that long-term exposure to high-intensity UV-A inhibits the growth of *Amaranthus tricolor*, and that removing 69 μmol·m^−2^·s^−1^ of UV-A from sunlight using a UV-blocking film resulted in higher biomass production over a 50-day study period (Kataria et al., 2013). In contrast to most previous studies that only quantify the responses at the end of the study, we harvested the plants periodically to track the changes in biomass, morphology, and phytochemical production in response to UV-A treatments. We observed that at three DAT, there were no significant differences in the shoot fresh and dry weight among all treatments. However, the biomass decreased in the treatments with higher UV-A after longer periods of exposure (Figure 2). A high dosage of UV-A (30 W·m^−2^) applied over a long period might have induced damage to DNA, proteins, and the photosynthetic apparatus (Diffey, 1991), eventually leading to a reduction in plant biomass.

The leaf area and size changes showed similar patterns as the plant biomass, with reduced growth under the UV-A 30 W·m^−2^ treatment at 14 DAT (Figure 3). Previous studies have shown that low levels of UV-A radiation from 9 to 35 μmol·m^−2^·s^−1^ (note that 35 μmol·m^−2^·s^−1^ of UV-A is equivalent to approximately 10 W·m^−2^ in our study) can promote biomass production as well as leaf expansion (Brazaitytė et al., 2010; Chen et al., 2019; Lee et al., 2019b; Zhang et al., 2020). The increase in leaf expansion under low/mild UV-A application may be mediated by the blue/UV-A-sensing photoreceptors cryptochromes and phototropins (Sakamoto and Briggs, 2002; Ohgishi et al., 2004; Kang et al., 2005; Mishra and Khurana, 2017; Mariz-Ponte et al., 2018). In contrast, high levels of UV-A irradiation may lead to cell necrosis or damage to the photosynthetic apparatus in plants (Mariz-Ponte et al., 2018; Lee et al., 2019b). Our results indicated that both the intensity and duration of UV-A exposure play an important role in biomass production and leaf morphological changes. Significant differences in biomass and leaf morphology could be detected at 7 DAT. The observed reductions in 30 W·m^−2^ UV-A treatment from seven DAT may have exceeded the threshold of beneficial UV-A dosage for basil growth.

### Photosynthetic Responses to Different UV-A Intensities

Prolonged exposure to high-intensity UV-A radiation has been reported to damage the PSII complex, leading to photoinhibition (Turcsányi and Vass, 2000; Vass et al., 2002). We observed contrasting results depending on the UV-A intensities. The P_net_ and g_s_ of basil under a high dosage of UV-A application (30 W·m^−2^) were significantly reduced at 14 DAT, but not at 3 and 7 DAT (Figures 4A,B). This indicates that, similar to leaf expansion and biomass production, the decreases in photosynthetic parameters were related to both high intensity and prolonged duration of UV-A exposure. A previous study reported no significant differences in the photosynthetic rates of kale after a short-duration UV-A application for 5 days at an intensity of 30 W·m^−2^ (Lee et al., 2019b). Another study reported that lettuce plants receiving UV-A radiation of 30 μmol·m^−2^·s^−1^ (≈10 W·m^−2^) for 13 days showed a decrease in *F_v_/F_m_*. However, P_net_ and g_s_ were not significantly different from plants receiving lower intensity UV-A (Chen et al., 2019). In this study, basil grown under mild UV-A treatment of 10 W·m^−2^ had significantly higher photosynthetic rate and stomatal conductance than those under the 20 and 30 W·m^−2^ treatments. Low UV-A light intensity might positively affect photosynthesis rates and stomatal conductance as UV-A photons can stimulate phototropins, causing stomatal opening (Christie et al., 2015; Isner et al., 2019).

The maximum quantum efficiency of PSII for photochemistry (*F_v_/F_m_*) showed a similar trend to the photosynthesis rate (Figure 4D). *The F_v_/F_m_* ratio in the 20 and 30 W·m^−2^ UV-A treatments gradually decreased over time, whereas that in the 10 W·m^−2^ treatment was maintained at a steady level similar to the 0 W·m^−2^ UV-A treatment. High-intensity UV-A can damage the D1 and D2 proteins in the reaction center of PS II (Greenberg et al., 1989; Christopher and Mullet, 1994) and eventually cause decreases in the maximum quantum efficiency of PSII photochemistry, electron transport rate, and photosynthesis due to the destruction of PS II reaction centers (Vass et al., 2002). Bidel et al. (2015) reported that excessive UV-A radiation could induce necrosis and disruption of the photosynthetic apparatus with a similar level of damage as under UV-B radiation. Such negative effects may be caused by an increase in reactive oxygen species (ROS), a decrease in Rubisco content and activity, and increased stomatal resistance (Takahashi and Badger, 2011; Nawkar et al., 2013; Kosobryukhov et al., 2015). Our results showed that mild-intensity UV-A (10 W·m^−2^) did not negatively affect photosynthetic responses. However, higher levels of UV-A at 30 W·m^−2^ for 14 days caused reductions in photosynthetic parameters. This indicates that exposure time and light intensity should be considered when using UV-A light as a potential beneficial stressor.

### Phenolic Compound Production in Response to UV-A Intensities

Plants produce diverse secondary metabolites as defense mechanisms under various abiotic stresses that cause ROS generation (Zhang and Kirkham, 1994; Rivero et al., 2001; Gill and Tuteja, 2010; Nikolaeva et al., 2010; Hasanuzzaman et al., 2012; Takshak and Agrawal, 2019). Previous studies found that UV-A irradiation, especially when applied at low intensities, can induce the accumulation of beneficial secondary metabolites, such as antioxidants, PAL enzyme, and phenolics. Additionally, it could improve biomass production or have no effect on biomass in various leafy greens and fruiting vegetables (Tsormpatsidis et al., 2008; Brazaityte et al., 2015; Soohyun et al., 2018; Chen et al., 2019; Lee et al., 2019b; Zhang et al., 2020). This study found that basil grown under the 20 W·m^−2^ treatment had the highest PAL enzyme activity, total phenolic concentration, and antioxidant capacity, followed by the 10, 30, and 0 W·m^−2^ treatments. Additionally, the treatment effects were greater at 14 DAT (Figure 5). For the 10 W·m^−2^ UV-A treatment, biomass production of basil and the accumulation of phenolic compounds were higher. This is similar to the improved biomass and total phenolic concentration observed in previous studies that used low UV-A intensities (under 35 μmol·m^−2^·s^−1^ or 10 W·m^−2^) or high UV-A light intensity with short exposure time (30 W·m^−2^ for 5 days). Although there were no significant differences in biomass production between the 0 and 30 W·m^−2^ treatments at 3 DAT, a significant increase in total phenolic concentration and antioxidant capacity was detected under the 30 W·m^−2^ treatment. However, at 7 DAT, the photosynthetic parameters under the 30 W·m^−2^ UV-A treatment gradually decreased, and at the end of the experiment (14 DAT), plants showed lower biomass production but a similar total phenolic concentration as those in the 0 W·m^−2^ UV-A treatment. In part, the decreases in biomass and phenolic compounds under the 30 W·m^−2^ UV-A treatment could be associated with the reductions in photosynthetic responses, including the *F*_v_/*F*_m_ ratio. In contrast, the mild UV-A treatment (10 W·m^−2^) had similar biomass as the 0 W·m^−2^ UV-A treatment and enhanced phenolic compound accumulation to a level similar to the 20 W·m^−2^ treatment (Figure 5). Note that we expressed the total phenolic concentration, PAL activity, and antioxidant activity on a fresh weight basis since sweet basil is commonly sold and consumed fresh. The percent shoot dry mass (shoot dry/fresh mass; %) among the UV-A treatments was nearly identical within each harvest date (Supplementary Table S2). Thus, the observed differences in those parameters among the UV-A treatments would remain the same when expressed on a dry weight basis. Our results indicate that high-intensity UV-A application of 30 W·m^−2^ in basil may be excessive and can cause a reduction in biomass production without enhancing the accumulation of beneficial phenolic compounds and antioxidants. Mild UV-A applied at 10–20 W·m^−2^ may be used as a beneficial stressor to enhance the nutritional quality and health-promoting benefits of sweet basil.

## Concluding Remarks

Our results indicated that high levels of UV-A radiation (30 W·m^−2^) applied over 14 days negatively affected the yield, photosynthesis, and accumulation of phenolic compounds in sweet basil. In contrast, mild UV-A treatment of 10 W·m^−2^ led to the highest biomass production and phenolic content without causing reductions in photosynthetic capacity (*F_v_/F_m_*). Thus, mild UV-A radiation (10–20 W·m^−2^) may be supplemented to improve both yield and quality of sweet basil produced in controlled environments such as greenhouses and indoor farms, where there is often a lack of UV radiation. The responses to UV-A radiation may depend on the background photosynthetic light intensity. Further studies on the potential interactive effects between UV-A dosage and the intensity of photosynthetic light might be needed to develop improved lighting strategies for producing high-quality basil.

## Data Availability Statement

The original contributions presented in the study are included in the article/Supplementary Material, further inquiries can be directed to the corresponding authors.

## Author Contributions

SK, JEK, and JK designed the experiments. SK and JEK performed the experiments. SK and SZ analyzed the data and wrote the first draft. SK, SZ, and JK discussed the data and revised the manuscript. All authors contributed to the article and approved the submitted version.

## Funding

This work was supported by the BK21 FOUR program (grant no. 4299991014324), funded by the National Research Foundation of Korea (NRF).

## Conflict of Interest

The authors declare that the research was conducted in the absence of any commercial or financial relationships that could be construed as a potential conflict of interest.

## Publisher’s Note

All claims expressed in this article are solely those of the authors and do not necessarily represent those of their affiliated organizations, or those of the publisher, the editors and the reviewers. Any product that may be evaluated in this article, or claim that may be made by its manufacturer, is not guaranteed or endorsed by the publisher.
