# Supplementary material for: Mild-Intensity UV-A Radiation Applied Over a Long Duration Can Improve the Growth and Phenolic Contents of Sweet Basil
Source: Front Plant Sci. 2022 Apr 18;13:858433. doi: 10.3389/fpls.2022.858433 (PMC9062229; doi:10.3389/fpls.2022.858433)
Supplement: Supplementary file 1 [file Table_1.pdf]

Table S1. Plant height, plant weight, leaf thickness, and soil plant analysis development (SPAD) of *Ocimum basilicum* under different UV-A light intensities (0, 10, 20, and 30 $W \cdot m^{-2}$; peak at 385 nm) combined with RGB LEDs (red, green, and blue peak at 664, 524, and 451 nm, respectively). Data points represent mean ± *SE* (n = 8; 2 plants per treatment × 4 replications). NS indicates non-significance at $P >= 0.05$.

Treatment	Plant height (cm)	Plant width (cm)	Leaf thickness (mm)	SPAD
UV 0 $W \cdot m^{-2}$	21.64 ± 0.33	18.74 ± 0.45	0.391 ± 0.006	44.29 ± 0.43
UV 10 $W \cdot m^{-2}$	21.81 ± 0.43	18.92 ± 0.51	0.392 ± 0.004	44.51 ± 0.78
UV 20 $W \cdot m^{-2}$	22.19 ± 0.46	20.04 ± 0.56	0.388 ± 0.004	43.57 ± 0.51
UV 30 $W \cdot m^{-2}$	21.75 ± 0.42	19.44 ± 1.07	0.400 ± 0.003	42.64 ± 0.90
Significance	NS	NS	NS	NS
