# Supplementary material for: Mild-Intensity UV-A Radiation Applied Over a Long Duration Can Improve the Growth and Phenolic Contents of Sweet Basil
Source: Front Plant Sci. 2022 Apr 18;13:858433. doi: 10.3389/fpls.2022.858433 (PMC9062229; doi:10.3389/fpls.2022.858433)
Supplement: Supplementary file 2 [file Table_2.pdf]

Table S2. Shoot dry/fresh weight (%) of *Ocimum basilicum* under four UV-A radiation treatments harvested at three time intervals. Data points represent mean ± SE (n = 8; 2 plants per treatment × 4 replications replications). NS indicates non-significance at $P >= 0.05$ within each harvest date.

	------------Days after Treatment (DAT)----------		
Treatment	3 DAT	7 DAT	14 DAT
UV 0 $W \cdot m^{-2}$	8.47 ± 0.47	8.77 ± 0.15	10.02 ± 0.23
UV 10 $W \cdot m^{-2}$	8.13 ± 0.38	8.77 ± 0.16	10.09 ± 0.23
UV 20 $W \cdot m^{-2}$	8.46 ± 0.22	8.90 ± 0.34	10.02 ± 0.34
UV 30 $W \cdot m^{-2}$	8.49 ± 0.24	8.71 ± 0.23	9.98 ± 0.38
Significance	NS	NS	NS
